# Philadelphia Letter

**Published:** 1894-09

**Authors:** 


### Correspondence.

### PHILADELPHIA LETTER.

[*Special correspondence of the Buffalo Medical and Surgical Journal.*]

*The American Academy of Medicine—The Abuse of Medical Charity in the form of Free Dispensaries—The Failure of Queen & Co., the well-known dealers in Optical Goods—Gifts to Medical Colleges and Hospitals.*

THE American Academy of Medicine has during the past decade thrown its powerful influence on the side of those who fought for higher medical education. The fight has been won, the aim is practically accomplished, and what is left to do will regulate itself without any special effort. Although we may yet be treated occasionally to a discussion on "the necessity for higher medical education" by those who have nothing original to offer to the profession, the work of creating a strong sentiment in favor of high standards is to a large extent accomplished, and he must be a courageous man indeed who will dare to stand up today and advocate a retrograde movement. It is very probable that the Academy will now, or in the near future, devote its energy to a subject of almost equal importance to the medical profession, although in a different sense, and that is the abuse of medical charity in the form of free dispensaries. The key-note of this abuse was probably struck by the *London Lancet* some time ago in an editorial on this question:

> The absence of any effective inquiry into the fitness of patients is monstrous. What would be thought of a poor-law system which gave relief without inquiry? It would be denounced as immoral and demoralizing. It is not otherwise with voluntary charities. Much efficiency may be dearly bought at the expense of the medical profession in the first place, and of demoralization of the poor in the second. The wide disapproval of it has been too long disregarded by hospital governors, and, we fear we must add, to some extent by medical teachers.

We have no reliable statistics in America relating to dispensary work, but, some years ago, reports gave to Edinburgh, with a population of 236,000, 43 per cent., *i. e.*, 103,100 who were relieved by medical charities. In Manchester the conditions were the same until the abuse received its proper attention and in ten years the 43 per cent. had been reduced to the reasonable figure of 6.89 per cent. There is not much doubt that we here in the United States are not much behind in this matter when statistics are compared, and if the Academy can find friends enough among the profession at large to bring this "charity racket with free lunch counter" in the form of free medicines to its proper level, as successfully and as speedily as the higher medical education question, then a large majority of the individual profession, especially its younger members, will rise and "call them blessed."

---

The well-known firm of Queen & Company, of this city, has failed, and it is interesting to note that one of the causes given to account for this failure is "a controversy with the medical profession of this city!" This "controversy" may find application in Buffalo. The firm, like almost all dealers in optical instruments, did, by an abundance of advertising, a thriving business in fitting people's eyes with spectacles on the plan of "gold glasses for $3.00 and upward, and eyes tested free of charge,"—just like they do it in Buffalo. Queen & Company, not satisfied with this, would employ at different times a recent graduate to do the testing, of course just as unscientifically as the optician's assistant did it before him, and, in consequence, failing to relieve just as many eyes, —but the halo of an M. D., though he had degraded himself voluntarily into an optician's clerk, drew large crowds to Queen & Company to have their eyes tested by a *physician*, "free of charge."

Finally, the medical profession of the city, recognizing the danger arising from this kind of performance, took the matter in hand, and after the "controversy," lasting quite a while, was ended, Queen & Co. ceased testing eyes for glasses altogether, and announced that they would only make spectacles from physician's prescriptions, thus finally recognizing the proper place of the optician in the community. The result shows that the income heretofore derived by the firm from misfitting the eyes of their fellow-men must have been large, indeed, if its loss was one of the causes of their failure.

---

Gifts and legacies to medical colleges and hospitals are so few and far between, that each one ought to be recorded all over the land. Mrs. Eva Muller, a Philadelphia lady, left $5,000 to the German hospital absolutely, another $5,000 to endow a "Fanny Muller Free Bed," and another $2,000 on the death of an old lady who has to have the use of it during her life-time. We may well say: Go and do likewise. John Jones may build for himself a costly mausoleum, costing hundreds of thousands, and the next generation has forgotten who he was and passes perhaps remarks upon his wretched taste when walking past the expensive pile ; but a free bed, a free hospital, a free laboratory, an endowed professorship, these things *live* forever and the donor is *never* forgotten.

